# Low genetic diversity of *Treponema pallidum* ssp. *pertenue* (TPE) isolated from patients’ ulcers in Namatanai District of Papua New Guinea: Local human population is infected by three TPE genotypes

**DOI:** 10.1371/journal.pntd.0011831

**Published:** 2024-01-02

**Authors:** Monica Medappa, Petra Pospíšilová, Maria Paula M. Madruga, Lucy N. John, Camila G. Beiras, Linda Grillová, Jan Oppelt, Arka Banerjee, Marti Vall-Mayans, Oriol Mitjà, David Šmajs

**Affiliations:** 1 Department of Biology, Faculty of Medicine, Masaryk University, Brno, Czech Republic; 2 National Department of Health, Aopi Centre, Port Moresby, Papua New Guinea; 3 Faculty of Medicine, University of Barcelona, Barcelona, Spain; 4 Department of Pathology and Laboratory Medicine, University of Pennsylvania, Philadelphia, Pennsylvania, United States of America; 5 Department of Mathematics and Statistics, Indian Institute of Technology, Kanpur, Uttar Pradesh, India; 6 Skin NTDs and STI section, Fight Infectious Diseases Foundation, University Hospital Germans Trias i Pujol, Badalona, Spain; 7 Lihir Medical Centre, Lihir Island, Papua New Guinea; 8 School of Medicine and Health Sciences, University of Papua New Guinea, Port Moresby, Papua New Guinea; Shandong Provincial Institute of Dermatology and Venereology, CHINA

## Abstract

Yaws is an endemic disease caused by *Treponema pallidum* subsp. *pertenue* (TPE) that primarily affects children in rural regions of the tropics. The endemic character of yaws infections and the expected exclusive reservoir of TPE in humans opened a new opportunity to start a yaws eradication campaign. We have developed a multi-locus sequence typing (MLST) scheme for TPE isolates combining the previously published (TP0548, TP0488) and new (TP0858) chromosomal loci, and we compared this typing scheme to the two previously published MLST schemes. We applied this scheme to TPE-containing clinical isolates obtained during a mass drug administration study performed in the Namatanai District of Papua New Guinea between June 2018 and December 2019. Of 1081 samples collected, 302 (28.5%) tested positive for TPE DNA, from which 255 (84.4%) were fully typed. The TPE PCR-positivity in swab samples was higher in younger patients, patients with single ulcers, first ulcer episodes, and with ulcer duration less than six months. Non-treponemal serological test positivity correlated better with PCR positivity compared to treponema-specific serological tests. The MLST revealed a low level of genetic diversity among infecting TPE isolates, represented by just three distinct genotypes (J_E_11, S_E_22, and T_E_13). Two previously used typing schemes revealed similar typing resolutions. Two new alleles (one in TP0858 and one in TP0136) were shown to arise by intragenomic recombination/deletion events. Compared to samples genotyped as J_E_11, the minor genotypes (T_E_13 and S_E_22) were more frequently detected in samples from patients with two or more ulcers and patients with higher values of specific TP serological tests. Moreover, the A2058G mutation in the 23S rRNA genes of three J_E_11 isolates was found, resulting in azithromycin resistance.

## Author summary

Yaws is an endemic disease that currently affects marginalized populations in thirteen countries. While the first yaws eradication effort in the 1950s resulted in a dramatic reduction of yaws cases, a resurgence of yaws has been observed in recent years. New treatment options including a single oral dose of azithromycin as well as new treatment regimens open a new opportunity to restart yaws eradication. The bacterium causing yaws, *Treponema pallidum* ssp. *pertenue* (TPE), has been characterized in this study with a newly developed multilocus sequence typing (MLST) scheme for TPE isolates collected during the Yaws 3 Trial held in the Namatanai District of Papua New Guinea. Altogether, 3 distinct TPE genotypes (J_E_11, S_E_22, and T_E_13) have been found as well as two new alleles of analysed loci that were shown to arise by intragenomic recombination/deletion events. Our typing method and meta-analysis may aid the ongoing yaws eradication campaign by distinguishing of infecting TPE strains to help control yaws transmission, monitoring antibiotic resistance emergence and patient reinfection in high-risk areas through targeted interventions.

## Introduction

Yaws is an endemic disease caused by *Treponema pallidum* (TP) subsp. *pertenue* (TPE) that primarily affects children in rural regions of the tropics and disseminates via direct skin contact with infected lesions [1]. Clinical manifestations in the primary stage include chronic skin ulcers and other skin lesions, potentially affecting bones and cartilage in the later stages [2].

The endemic character of yaws infections in Africa, southern Asia, and the Pacific region [3] and the believed exclusive reservoir of TPE in humans opened an opportunity for a yaws eradication campaign known as the Morges strategy [4]. The first yaws eradication effort in the 1950s resulted in a dramatic reduction (~95%) in yaws cases. Since then, sporadic yaws outbreaks have been reported in Papua New Guinea, with clusters of cases concentrated in several provinces and including imported yaws cases [5]. Recently, new treatment options, including a single oral dose of azithromycin, have been successfully tested [6,7], as have other treatment regimens [8].

One newly tested treatment option includes round(s) of total community treatment (TCT) followed by total target treatment of active cases (TTT). While several rounds of TCT were superior to one round of TCT followed by several TTT rounds [8], this approach benefits from molecular strain TPE typing to help discriminate the epidemiological sources of reintroduced infections and/or treatment failures due to emerging antibiotic resistance.

Molecular typing provides population structure data that can map the geographical distribution of strain types, draw associations between strains and specific demographic groups, and examine transmission dynamics. In some cases, molecular typing can also be a valuable diagnostic tool, as illustrated by recent syphilis-bejel misdiagnoses [9,10].

Historically, there were three significant attempts to develop typing method of TPE, including a TPE MLST scheme based on the characterization of TP0136, TP0326, and TP0548 loci [11] and a scheme based on testing TP0488 and TP0548 loci [12] and application of CDC syphilis typing scheme to yaws. While the first scheme detected seven genotypes among 194 samples from yaws patients on Lihir Island, Papua New Guinea [11], the second scheme differentiated 31 genotypes out of 59 fully typed TPE samples isolated from non-human primates (NHP) in Africa [12]. In addition, CDC typing scheme [13] analyzed the number of 60-bp tandem repeats in the arp (acidic repeat protein) gene (TP0433), fragment length polymorphism of the 3 simultaneously PCR-amplified tpr genes (tprEGJ), 84 nt-long sequence of TP0548 and guanine homonucleotide tandem repeat upstream of the *rps*A coding sequence. This typing resulted in discovery of 2 genotypes among clinical 14 samples from Vanuatu and 9 genotypes from clinical 16 samples from Ghana [13].

In this study, we tested an MLST typing scheme based on three loci: (1) TP0548 locus used by both studies [11+12], (2) TP0488 used in a study by Chuma et al. (2019) [12], and (3) the TP0858 locus (used in this study). In addition, samples from this study were typed by previous schemes and compared to the results of the previous studies.

## Material and methods

### Ethics statement

All the trial participants (for children, their parents, or guardians) provided oral informed consent for screening and treatment. The study protocol was approved by the Medical Research Advisory Committee of the Papua New Guinea National Department of Health (MRAC No: 17.19), which authorized oral consent [8].

### Collection of samples

Samples originated from patients living in the Namatanai District of Papua New Guinea. The samples were collected during the Yaws 3 Trial conducted over 1.5 year period between June 2018 to December 2019 [8]. Swab samples were collected from patients with ulcerative or nodular skin lesion on the lower limbs in June 2018 (first round), December 2018 (second round), June 2019 (third round) and December 2019 (fourth round). Samples were stored in a lysis buffer (100 mM TRIS, pH = 8; 100 mM EDTA, pH = 8; 1% SDS) and transported from Papua New Guinea to the Czech Republic.

### Selection of typing loci

The selection of typing loci is described in detail in the S1 Text). In summary, 10 complete and 13 draft TPE genomes were used to screen for the most variable loci and number of SNVs per kbp was assessed for these regions. Comparison of whole genome ML trees and trees of 6 most variable loci were used to assess the resolution power of individual loci for typing. Several selection criteria were therefore applied to obtain the most suitable loci for TPE typing, and these included (1) the percentage of genome-wide data resolution, (2) the number of SNVs per kbp, (3) the ability to distinguish TPE from TPA/TEN and TPE from TPA and TEN (Table D in S1 Text).

### DNA isolation and definition of typed genomic regions

Whole DNA from swab samples was extracted using QIAamp DNA mini kits (Qiagen, Hilden, Germany) according to the manufacturer’s instructions. TPE positivity was assessed by PCR amplification of the *pol*A (TP0105) target [14]; samples were considered PCR-positive when at least one additional TPE locus tested PCR-positive (from TP0488, TP0548, and TP0858, and 23S rDNA loci). MLST was performed following the amplification of three typing targets, including TP0488, TP0548, and TP0858. Based on the TPE Samoa D whole genome reference [15], regions between coordinates 522711 and 523867 (1157 bp), 593225 and 594283 (1059 bp), and 936024 and 937039 (1016 bp) were amplified using a two-step protocol (nested PCR) and then sequenced.

### Nested PCR used in MLST and PCR conditions

The first step of the nested PCR protocol was performed using PrimeSTAR GXL polymerase (Takara Bio Inc., Otsu, Japan) in a total volume of 25 μl consisting of 5× PrimeSTAR GXL buffer (5 μl), PrimeSTAR dNTP mix (2 μl), 10 pmoles of each primer and PrimeSTAR GXL DNA polymerase (0.5 μl), tested DNA (1 μl), using the following cycling conditions: 94°C initial denaturation for 1 minute, 8 cycles at 98°C (10 s), 68°C (15 s) with touch down protocol of −1°C per cycle and 68°C (1 m 45 s), followed by 35 cycles at 98°C (10 s), 61°C (15 s) and 68°C (1 m 45 s), with a final extension of 68°C (7 m). The second step was performed using *Taq* polymerase (New England Biolabs, Frankfurt am Main, Germany) in a total volume of 25 μl consisting of 10× Standard *Taq* reaction buffer (2.5 μl), 100 mM dNTPs with a final concentration of 200 μM, (0.5 μl), 25 pmoles of each primer, *Taq* DNA polymerase (0.1 μl) and tested DNA (1 μl): 94°C initial denaturation for 1 m, 40 cycles at 94°C (30 s), 48°C (30 s), 72°C (1 m 45 s) and a final extension at 72°C for 7 minutes. A list of primers used to amplify the typing loci is included in Table E in S1 Text.

### DNA sequencing and analysis

MLST amplified PCR products were Sanger sequenced by Eurofins Genomics (Constance, Germany; Eurofins Genomics Company). The chromatogram files (.ab1 files) were trimmed on either side to eliminate primer sequences and low-quality sequencing data. The data for each MLST locus were aligned against the Samoa D reference sequence of the corresponding locus (TP0488, TP0548, and TP0858). The sequencing reads were edited using Lasergene’s EditSeq sequence editor program (DNASTAR Lasergene EditSeq v.7.1.0; DNASTAR, Madison, WI, USA), consensus sequences were generated using the Seqman sequence assembling program (Lasergene, DNASTAR v.7.1.0). Sequences of allelic variants found in this study were submitted to Genbank under accession numbers OR509506-OR509513.

### Serological surveys

A 10 μL of capillary blood from participants of the study were obtained to perform a rapid quantitative serological test (dual-path platform [DPP] syphilis screen and confirm assay; Chembio Diagnostics, Medford, NY, USA) [8]. Naked eye and optical density microreader DPP values were recorded. A participant was considered to have positive yaws serology if the density of the treponemal line was greater than 12 (as established by the manufacturer).

### Statistical analysis

Demographic and clinical characteristics, as well as serological data, were tested for possible associations using Pearson’s chi-square test (IBM SPSS Version 29.0.0.0). Variables with numerical values were converted to categorical values. A goodness-of-fit model was used to compare the observed frequencies with the expected frequencies. P values ≤ 0.05 was considered statistically significant.

## Results

### Characteristics of the study participants

During the Yaws 3 Trial, 1081 swab samples were collected and analyzed for the presence of treponemal DNA [8]. The relevant characteristics of the study participants were collected for 1002 out of 1073 participants. Not all characteristics were provided for each individual and the summary of data is shown in Table 1. Most of the participants were under 15 years old and had single or multiple ulcers together with positive syphilis serology.

**Table 1:** Characteristics of the study participants. The patient’s characteristics were not available for all individuals (only for 1002 out of 1073).

Characteristics of study participants (n = 1002)	No. of study participants
Gender (M | F)	523 | 459*
Age (up to 9y | 10-15y | more than 15y)	364 | 313 | 298
No. of ulcers (1–2 | more than 2)	812 | 105
Size of ulcers (less than 2 cm | 2 cm | more than 2 cm)	343 | 253 | 286
Duration of ulcers (0–3 months | more than 3 up to 6 months | more than 6 months)	796 | 64 | 45
Ulcer episode (first | multiple)	752 | 206
Results of T-line serology (Positive | Negative)	275 | 693
Results of NT-line serology (Positive | Negative)	275 | 693
Results of T-line serology (up to 29 | 30–60 | more than 60)	449 | 97 | 274
Results of NT-line serology (up to 29 | 30–60 | more than 60)	515 | 97 | 208

*The number indicates patients for which the characteristics were known [8] as not all the characteristics were known for all patients. T-line serology–treponemal serology. NT-line serology–non-treponemal serology. Assessed by dual path platform syphilis screen and confirm assay on a finger capillary blood.NT line density thresholds ≥ 30 and ≥ 90 correspond to rapid plasma reagin 1:4 and 1:16, respectively.

The detailed selection of MLST typing loci is described in the S1 Text and supported by analysis of genome sequences available until 2018. The selection of the loci was driven by the aim to design typing system which captures the highest level of diversity based on the available TPE genomes at the moment of the study design. With this approach, we propose a new MLST scheme for TPE strains based on sequencing three variable loci (TP0488, TP0548, and TP0858). Natural selection test detected positively selected sites also in the loci selected for the new MLST scheme (Table F in S1 Text) similarly to MLST in TPA [16]. However, testing of the selected loci revealed genetic stability of TPA during continuous rabbit propagation for 142 days [16].

### MLST of TPE isolates from Namatanai, Papua New Guinea

A total of 1081 swab samples were collected and analyzed for the presence of treponemal DNA. Samples were considered TPE positive when two independent TPE loci were positive during nested PCR amplifications, i.e., having positive *pol*A locus (TP0105) and at least one of the four tested loci, i.e., TP0488, TP0548, TP0858, or 23S rDNA. Of 1081 samples, 302 (28.5%) tested positive for TPE DNA, and 255 (23.5%) were fully typed. The TP0488, TP0548, and TP0858 amplicons were considered fully typed when the DNA sequence was determined for the entire amplicon without the corresponding primer and adjacent sequences, i.e., in 1117, 1018, and 976 nt-long regions for TP0488, TP0548, and TP0858, respectively. The coordinates of the evaluated sequences corresponded to the following TPE Samoa D coordinates: 522731 to 523847 (1117 bp), 593246 to 594263 (1018 bp), and 936044 to 937019 (976 bp).

### Allelic variants of typing loci TP0548, TP0488 and TP0858

**Fig 1:**
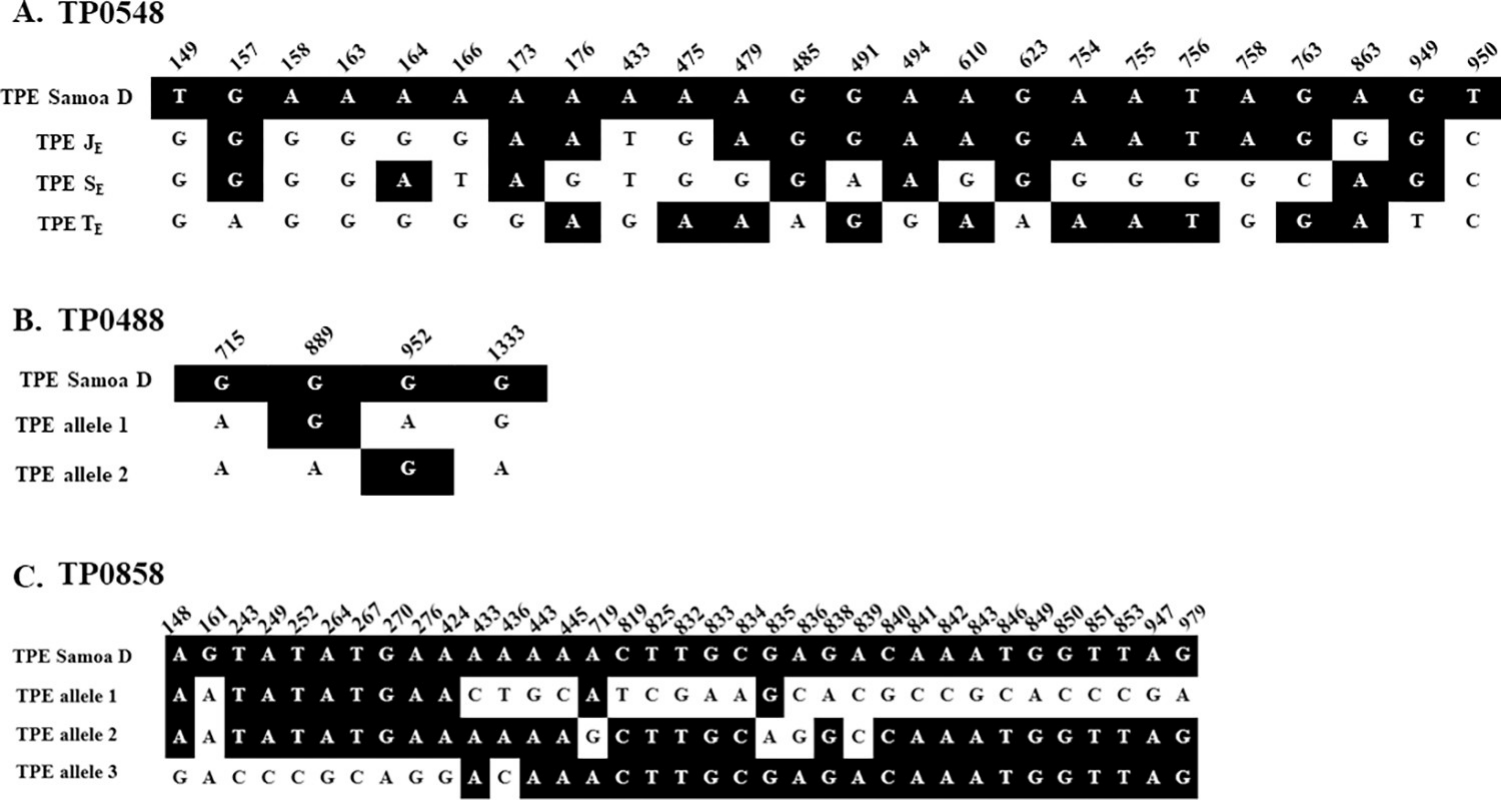
Sequence differences between alleles detected among TPE-containing samples from Namatanai, Papua New Guinea. The S_E_22 genotype is highly related to the sequence of TPE Samoa D, a strain isolated initially in Polynesia in 1953 [17] and sequenced later [15]. The T_E_ allele was divergent from TPE Samoa D and TP0858 allele 3. The TP0858 allele 3 appears to result from the intragenomic reshuffling of the TP0856 locus, a mechanism suggested earlier [18]. The predominant J_E_11 genotype resembles the TPE Samoa D sequence except for allele TP0858, which was described as recombinant in the Indonesian TPE strain Kampung Dalan K363 [18]. The numbers above nucleotide positions indicate gene coordinates in the TPE Samoa D.

While three allelic variants (J_E_, S_E_, T_E_, and 1, 2, 3, respectively) were determined in the TP0548 and TP0858 loci, only two allelic variants (1 and 2) were found in the TP0488 locus. The allelic variants of different loci did not freely combine, except for TP0488 allele 1, which was associated with both the J_E_ and T_E_ TP0548 alleles and with alleles 1 and 3 of TP0858. The differences between alleles are shown in Fig 1.

Fully typed samples analyzed in this study were distributed as follows, the first round of sample collection (n = 163, 32%), the second round (n = 7, 6.3%), the third round (n = 35, 18.8%), and the fourth round (n = 50, 17.9%) [8]. Three different genotypes were identified in the 255 fully typed samples. The results of the MLST typing are shown in Table 2. While alleles in the TP0488 and TP0858 loci were designated by numbers, we retained the original designation of the TP0548 locus using letters [11]. Moreover, the genotype descriptions start with TP0548 (and not with TP0488) in order to resemble the scheme introduced by Godornes et al. (2017) [11]. Since we sequenced a larger region of the TP0548 locus than the previous study [11], we added a subscript index (_E_), indicating that the sequence was determined in an extended locus (e.g., J_E_, extended allele J).

**Table 2:** MLST typing of TPE-containing isolates from Namatanai, Papua New Guinea.

Genotype	TP0548 allele	TP0488 allele	TP0858 allele	Percentage of genotypes from all typeable samples (n = 255)
**J**_**E**_ **11**	J_E_*	1	1	93.3% (n = 238)
**S**_**E**_ **22**	S_E_	2	2	4.3% (n = 11)
**T**_**E**_ **13**	T_E_	1	3	2.0% (n = 5)
**J**_**E**_ **11/T**_**E**_ **13**	J_E_/T_E_	1	1/3	0.4% (n = 1)

*J_E_, sequence corresponds to the previously determined allele J [11], but the sequence is determined in an extended locus (J_E_, extended). J_E_11 corresponds to the previously described genotype J11 [8].

The most predominant TPE genotype circulating in Namatanai was J_E_11 followed by S_E_22 and T_E_13. In a sample (T04.ba.79) isolated from a single patient, DNA corresponding to two TPE genotypes (J_E_11 and T_E_13) was detected.

### Geographic localization of detected genotypes and patient characteristics

**Fig 2:**
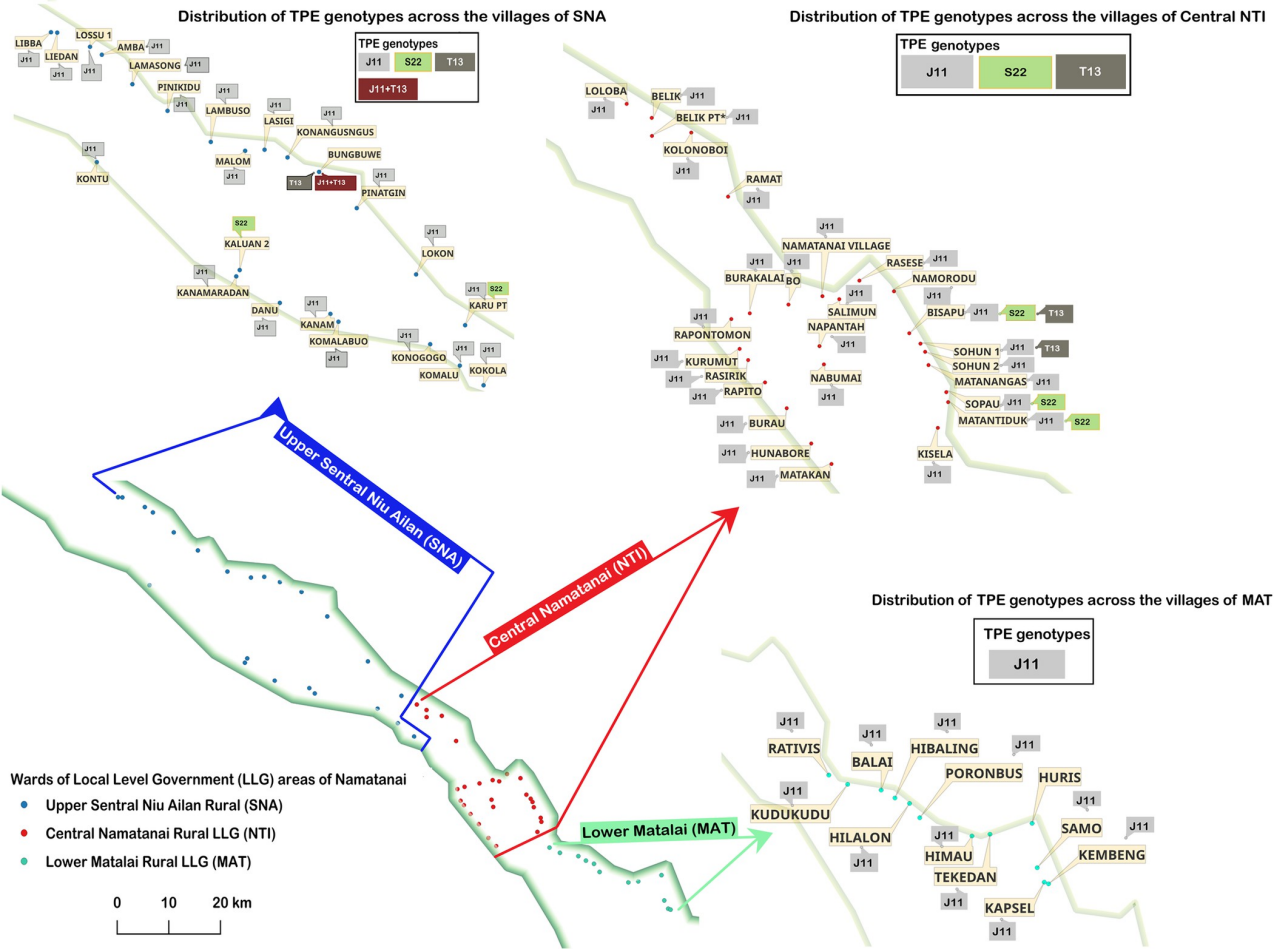
Geographical locations of detected genotypes in sampled wards in Namatanai, Papua New Guinea. The map is based on information from 164 participants from the first round. The minor T_E_13 genotype was only detected in two neighboring wards in the central part of the sampled Namatanai District and in one patient who was coinfected with both J_E_11 and T_E_13 genotypes from the northern part. The S_E_22 genotype was detected in four individual wards relatively distant from each other. This map was constructed using QGIS 3.28.0, baselayer made with Natural Earth. Free vector and raster map data @ naturalearthdata.com.

As described previously [8], patient samples were collected in individual wards (n = 38). Their location in three local-level government (LLG) areas of the Namatanai District is shown in Fig 2. The most predominant TPE genotype J_E_11 was detected in all wards, while the S_E_22 was found in two wards in the north and center of the sampled region. The minor T_E_13 genotype was detected in two wards in the central region of the sampled area. In addition, one sample from northern ward number 11 (Bungbuwe, SNA) contained DNA of both J_E_11 and T_E_13 genotypes.

### Association of TPE PCR-positivity with patient characteristics

The analysis of TPE PCR-positivity with patient’s characteristics, including age, gender, and clinical data, revealed significantly higher PCR-positivity in younger patients (Table 3), in patients with single ulcers of any size during first ulcer episode, and with ulcer durations less than six months. Moreover, non-treponemal serological test positivity correlates better with PCR positivity than treponema-specific serological tests (Table 3). In addition, PCR positivity correlated with the quantitative results of both non-treponemal and treponemal serological tests, being more frequently positive in patients with non-treponemal serological test positivity higher than 60 (61.1% of PCR positive samples). Compared to samples genotyped as J_E_11, the minor genotypes (T_E_13 and S_E_22) were more frequently detected in samples from patients with two or more ulcers, longer-lasting ulcers, and patients with higher values of specific TP serological tests (T-line).

**Table 3:** Association of TPE PCR-positivity and TPE genotypes with patient characteristics.

Characteristics	No. of PCR-negative samples (%)	No. of PCR-positive samples (%)	*p* value	No. of J_E_11 samples (%)	No. of T_E_13 and S_E_22 samples (%)	*p* value
**Gender** (M/F) (n = 977) *	359/340 **69.2/74.2**	160/118 **30.8/25.8**	0.08	123/90** 93.2/93.8	9/6 6.8/6.3	0.8
**Age** (up to 9y/10-15y/more than 15y) (n = 970)	217/222/254 **60.1/70.9/85.8**	144/91/42 **39.9/29.1/14.2**	<0.001	118/66/27 **95.9/93.0/84.4**	5/5/5 **4.1/7.0/15.6**	0.06
**No. of ulcers** (1/2/3 and more) (n = 912)	459/103/88 **69.0/72.5/83.8**	206/39/17 **31.0/27.5/16.2**	0.007	156/33/11 **94.0/97.1/78.6**	10/1/3 **6.0/2.9/21.4**	0.05
**Size of ulcers** (less than 2 cm/2 cm/more than 2 cm) (n = 877)	108/162/350 **77.1/64.0/72.3**	32/91/134 **22.9/36.0/27.7**	0.012	67/68/59 **89.3/91.9/98.3**	8/6/1 **10.7/8.1/1.7**	0.12
**Duration of ulcers** (0–3 mo, >3 mo up to 6 mo, >6 mo) (n = 900)	565/35/35 **71.4/54.7/77.8**	226/29/10 **28.6/45.3/22.2**	0.010	174/22/5 **93.5/95.7/83.6**	12/1/1 **6.5/4.3/16.7**	0.5
**Ulcer episode** (first/multiple) (n = 953)	513/167 **68.7/81.1**	234/39 **31.3/18.9**	<0.001	179/30 **93.7/93.8**	12/2 **6.3/6.3**	0.99
**Serology T-line** (positive/negative) (n = 967)	349/344 **61.7/85.8**	217/57 **38.3/14.2**	<0.001	166/45 **91.7/100**	15/0 **8.3/0**	0.046
**Serology NT-line** (positive/negative) (n = 967)	273/420 **54.3/90.5**	230/44 **45.7/9.5**	<0.001	179/31 **92.3/100**	15/0 **7.7/0**	0.10
**Serology T-line** (less than 30/30-60/more than 60) (n = 815)	375/58/145 **83.5/63.0/52.9**	74/34/129 **16.5/37.0/47.1**	<0.001	57/26/91 **98.3/100/86.7**	1/0/14 **1.7/0/13.3**	0.009
**Serology NT-line** (less than 30/30-60/more than 60) (n = 815)	448/49/81 **87.5/51.6/38.9**	64/46/127 **12.5/48.4/61.1**	<0.001	44/34/96 **95.7/89.5/91.4**	2/4/9 **4.3/10.5/8.6**	0.544

*The number indicates patients for which the characteristics were known [8].

**The patient characteristics were not available for all genotyped isolates

### The modular structure of TP0858 alleles

**Fig 3:**
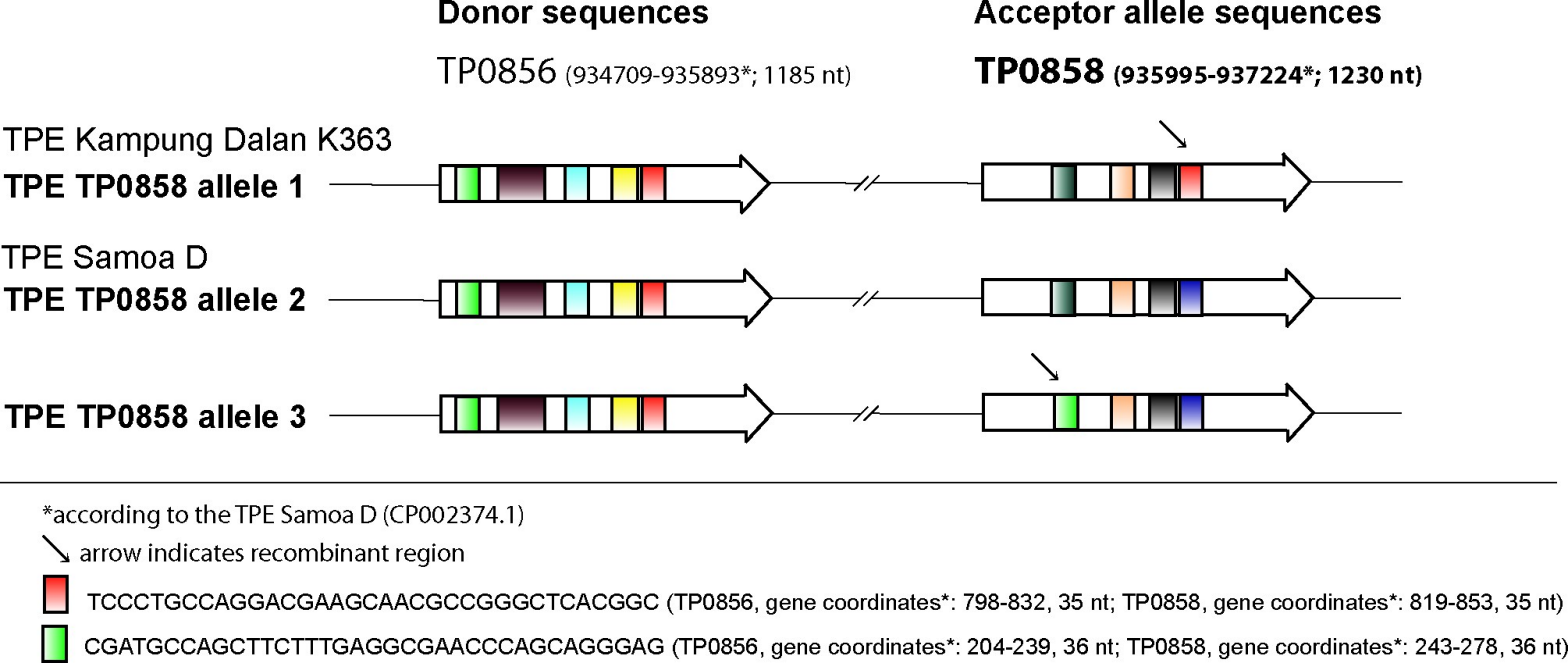
Modular structure of TPE TP0858 alleles 1, 2, and 3 from TPE-containing samples from Namatanai, Papua New Guinea. While TP0858 allele 2 has a modular structure similar to TPE Samoa D, TP0858 allele 1 has a similar modular structure as Kampung Dalan K363 [18]. The TP0858 allele 3 represents a novel allele with a unique modular structure. The arrows denote recombinant regions. The figure was modified according to Strouhal et al. (2018) [18], and an additional donor site is shown. All sequences shown correspond to the Samoa D sequences, while the sequences determined in this study differ in several nucleotide positions. Not all identified donor sites were found among TPE strains and isolates.

As described previously, the TP0858 gene has a modular structure, where sequences from the TP0856 locus are copied to the TP0858 locus via gene conversion [18]. The TP0858 allele 1 was found to be very closely related (differing in just seven single nucleotide differences) to the allele present in the Indonesian strain Kampung Dalan K363 representing recombinant allele [18] and discovered in a portion of strains sequenced by Marks et al. (2018) [19] from the Solomon Islands. The TP0858 allele 2 was similar to the allele found in Indonesian strain Sei Geringging K403 and Samoa D (3 and 5 single nucleotide differences, respectively). The TP0858 allele 3 represents a new recombinant allele, and its modular structure is shown in Fig 3.

### Macrolide resistance mutations in the 23S rRNA genes

Although an analysis of both 23S rRNA genes was not part of the MLST scheme, the previously described mutations leading to macrolide resistance, A2058G and A2059G in the 23S rDNA [20,21], were evaluated. Among 255 fully typed samples, we detected three macrolide-resistant patients carrying mutation A2058G in fourth round (18 months post total community treatment by azithromycin). All three patients were infected with the same TPE genotype (J_E_11) and were epidemiologically related (2 brothers and a classmate of one of the brothers).

### Comparison of the new MLST typing scheme with the TP0136, TP0326, and TP0548 scheme and with the CDC typing scheme

**Fig 4:**
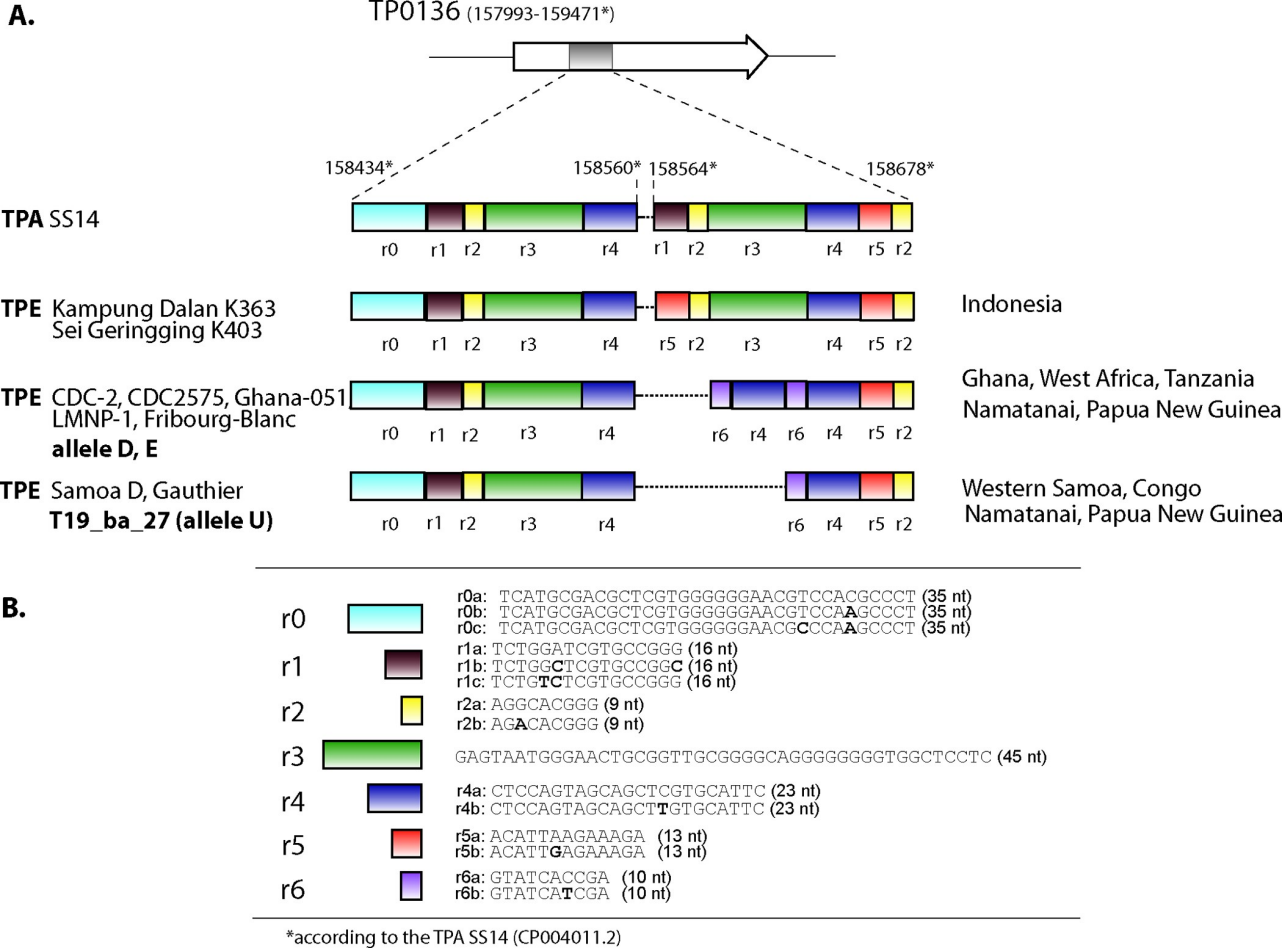
**A.** Modular structure of TPE TP0136 alleles D, E, and U from TPE-containing samples from Namatanai, Papua New Guinea. Both alleles D and E (shown in bold) have identical modular structures with TPE CDC-2, CDC2575, Ghana-051, LMNP-1, and Fribourg-Blanc; however, both alleles D and E differ in several individual nucleotide positions. Allele U (shown in bold) has a different modular structure identical to TPE Samoa D and Gauthier. For comparison, the modular structure of TP0136 from Indonesian TPE strains Kampung Dalan K363 and Sei Geringging K403 and TPA strain SS14 have different modular structures [18]. **B.** Sequences of individual repeat motifs. Please note that the r0 motif does not repeat in TPE but was found repetitive in TPA TP0136 from a clinical isolate from the Czech Republic (Allelic profile: 18.1.1; [24]). The figure was modified according to Strouhal et al. (2018) [18].

A comparison of the new MLST typing scheme analyzing TP0548, TP0488, and TP0858 with previously published schemes was performed by additional sequencing of TP loci in the available TPE isolates according to published protocols [11,13]. The TP0136 and TP0326 loci were sequenced for 21 and 214 samples, respectively, and the sequences were analyzed with respect to identified alleles. In general, both typing schemes showed similar typing resolution (Table 4) except for a single isolate (T19.ba.27) where the previously developed typing scheme [11] identified TPE genotype S_E_22 with an unexpected TP0136 allele resembling the corresponding allele in TPE Samoa D and Gauthier [22]. In contrast, all other tested isolates and previously published alleles had a modular structure similar to TPE CDC-2 [16,23]. We named this allele U (unique) (Fig 4).

The comparison of our new MLST typing scheme with the previously introduced CDC typing scheme [13] was performed by analyzing the number of 60-bp tandem repeats in the *arp* (acidic repeat protein) gene (TP0433) and fragment length polymorphism (RFLP) analysis of the three simultaneously PCR-amplified *tpr* genes (*tpr*EGJ). Both typing schemes are compared in Table 4. The CDC typing on selected TPE isolates identified three types, 3q, 4q, and 5q, revealing that the CDC typing resolution was identical to our typing scheme.

**Table 4:** Comparison of a new MLST typing scheme with the previous MLST and CDC typing schemes.

MLST scheme (this study)	MLST scheme (Godornes et al., 2017) [11]	Number of analyzed samples	CDC typing scheme	Number of analyzed samples
**J**_**E**_ **11**	JG8	238	4q	53
**S**_**E**_ **22**	SE7/SU7*	10/1	5q	9
**T**_**E**_ **13**	TD6	5	3q	2
**J**_**E**_ **11/T**_**E**_ **13**	JG8/TD6	1	4q/3q	1

*The SU7 genotype contains a unique allele (U) in TP0136, resembling the corresponding allele in TPE Samoa D and Gauthier [15].

## Discussion

In this study, we developed a new MLST typing scheme for clinical TPE isolates by combining previously used typing loci TP0488 [12] and TP0548 [11] with a new locus TP0858. Under optimal conditions, the selected typing loci in bacterial MLST systems are highly diverse and have genetically stable chromosomal regions, which reduces the need for analyzing multiple loci. The conventional MLST approach, used in many bacterial species, types several housekeeping genes to avoid genetically highly diverse (and therefore unstable) loci. However, since *Treponema pallidum* is a monomorphic (genetically uniform) species without efficient horizontal transfer mechanisms, the existing MLST loci include either genes encoding outer membrane components (TP0548, TP0326, TP0136, TP0858) or genes coding for cytoplasmic and periplasmic proteins (TP0488, TP0705). The recently introduced high-resolution MLST scheme for syphilis treponeme (TPA) uses three loci (TP0136, TP0548, and TP0705) and has discriminatory power of about 30% compared to whole genome sequences [16]. However, this typing scheme appears to be suboptimal for TPE isolates since individual chromosomal loci appear to have different genetic variability in different *Treponema pallidum* subspecies (TPA, TPE, TEN; [25]) and therefore, the MLST typing schemes should reflect the individual treponemal subspecies to provide the maximum resolution and ability to discriminate among TPA, TPE, and TEN subspecies. In this study, we selected the loci with highest percentage of genome-wide data resolution, the number of SNVs per kbp, and the ability to distinguish TP subspecies using the minimal number of typing loci. Our typing scheme, based on analysis of TP0488, TP0548, and TP0858, revealed 70% of the whole genome resolution just by analyzing a 3,111 bp-long chromosomal sequence and was able to distinguish among TPA, TPE, and TEN infections. As shown previously by whole genome sequencing [26], the JG8 genotype (identical to J_E_11) comprised five sublineages, including JG8.c1, JG8.c2, JG8.c3, JG8.c4, and JG8.c5, showing that additional genetic diversity is present among J_E_11 genotypes, at least among Polynesian and Oceanian TPE isolates. However, J_E_11 samples from Lihir Island, PNG, differ in only a handful of nucleotide positions, further supporting the high resolution power of the new MLST scheme.

We applied this scheme to TPE-containing clinical isolates obtained in the Yaws 3 Trial in Namatanai District, Papua New Guinea, between June 2018 and December 2019 [8]. The TPE genotypes in Namatanai resembled Polynesian TPE strain Samoa D [15] and Indonesian TPE strains [18] rather than African TPE strains or isolates.

Of 302 positive TPE DNA samples, 255 (84.4%) were fully typed. Three different genotypes were found, one dominant and two minor genotypes. This genotype number is surprisingly low compared to a previous study on Lihir Island (between 2013–2016), a district close to Namatanai, Papua New Guinea, which after analyzing 194 completely typed samples [11], identified seven different genotypes. On the other hand, the number of identified alleles found in a study by Godornes et al. (2017) [11] was similar to our study and included 3, 3, and 3 alleles in the TP0548, TP0136, and TP0326 loci, respectively. However, unlike in our study, the detected alleles appeared to combine, e.g., allele J (TP0548) combined with alleles G (TP0136) and D (TP0136). In addition, the genotypes with different individual alleles on three chromosomal loci comprised the majority (97%) of the identified genotypes, suggesting that independent allele combinations are rare. Moreover, later work on whole genome sequencing of these samples [26] confirmed the presence of genotypes differing at all three loci, which, together with our data, suggest that TPE genotypes evolved simultaneously in all loci of the entire chromosome, thus precluding independent combination of alleles in MLST.

The incompletely typed samples comprised 47 of 302 PCR-positive clinical isolates (15.6%) in this study, and this number is comparable with other studies [11]. The reasons for incomplete typing are likely either a low treponemal count in specific swab samples or partial degradation of DNA in the sample or both. As shown previously, the PCR-positivity of TPA samples depended on the length of the PCR product in the first step of the PCR reaction [27], which is consistent with the degree of DNA fragmentation in the sample. Therefore, the shortest amplicons (e.g., *pol*A, [28]) have the highest detection rates.

TPE PCR-positivity was found higher in younger patients, in patients with single ulcers during the first ulcer episode, and with ulcer duration less than six months, suggesting that older patients, patients with multiple ulcers and repeated ulcerations together with long-lasting ulcers are either entering a latent phase of yaws where there are minimal numbers of treponemes in primary lesions or have an ulceration with different etiology than a typical TPE infection.

Non-treponemal serological test positivity correlated better with PCR positivity than treponema-specific serological tests. Since non-treponemal serological tests indicate the activity of treponemal infections, this finding was not surprising. Moreover, a clear correlation between quantitative results of a non-treponemal serological test with PCR positivity suggests that the number of treponemes (and therefore the extent of infection) is of crucial importance in the detection of treponemal DNA and that there could be potential swab samples where an insufficient amount of treponemal DNA resulted in negative TPE PCR detection despite the presence of TPE bacteria.

While the predominant TPE genotype J_E_11 was detected in all wards of sampled Namatanai District, minor S_E_22 and T_E_13 genotypes were detected only in specific areas (wards), suggesting that local contacts are essential in yaws transmission. Similar geographical clustering among TPE isolates has been previously noticed in African non-human primates [12]. The small number of detected TPE genotypes was surprising but consistent with the endemic character of yaws, suggesting infrequent (if any) introduction of new TPE strains into the community, which is in sharp contrast to TPA genotype variability in the infected human population [16,24,27,28,29]. The conducted study covered a geographical area of approximately 2400 square kilometers, which might contribute to the low detected level of genetic diversity. On the other hand, the low level of genetic diversity could be considered beneficial for yaws eradication efforts. The fact that the minor genotypes (T_E_13 and S_E_22) were more frequently detected in samples from patients with two or more long-lasting ulcers and from patients with higher TP-specific serological tests values could point to the presence of important differences in the infectivity/pathogenicity of individual TPE genotypes. The detected genetic differences along with additional possible genomic diversity among the three identified genotypes in this study could have functional consequences including changes in gene expression, differences in protein activities, and also differences in infectivity and pathogenicity.

Selected samples (n = 65) analyzed in this study were also typed using the CDC typing scheme [13], which analyzed the restriction profile of *tpr*EGJ genes and the number of repetitions within the *arp* gene (TP0433). CDC typing revealed three types, one type of *tpr* allele (called “q”) and three *arp* repetition number variants (3, 4, and 5); this resolution was identical to our MLST typing scheme.

Moreover, CDC types 4q, 5q, and 3q perfectly matched J_E_11, S_E_22, and T_E_13 allelic profiles, respectively. Compared to TPA, the TPE isolates appear to have only a limited number of *tpr* variants. In TPA isolates, these genes are under positive selection [25], and over two dozen RFLP variants have been described in TPA clinical isolates [30,31]. Further, *tpr*EGJ gene restriction profiles were shown to be variant (this was also true for parallel TPA samples taken from the same patient [32,33,34]), suggesting high variability of these loci among TPA clinical isolates. Similar to *tpr*EGJ genes in TPE, the numbers of repetitive motifs in the human TPE *arp* gene [23] are usually limited to low numbers and, therefore, quite different from TPA *arp* numbers [35]. Moreover, *arp* repeat motif numbers in TPE negatively correlate with the number of repetitive motifs in the TP0470 gene [35], which is not the case in human syphilis isolates where there are higher numbers of repetitions and no correlation with repeats in the TP0470 gene [35].

Two alleles (1 and 3) of TP0858 showed a different modular character of the TP0858 gene than the third allele (number 2) and the reference TPE Samoa D. As predicted previously, sequences from the TP0856 locus are being copied to the TP0858 locus via gene conversion [18]. While TP0858 allele 1 was found to be identical to the allele present in the Indonesian strain Kampung Dalan K363 [18], TP0858 allele 3 represents a novel intragenomic recombination event that adds a novel recombination sequence to the compendium of recombinant loci [22,25]. Both TP0856 and TP0858 genes are highly transcribed (and co-transcribed) in TPA Nichols [36,37,38] during experimental infection, suggesting that they have an essential role in treponemal pathogenesis, which is further supported by the positive selection of TP0858 variants in TPE [25].

During this study, an A2058G mutation in the 23S rRNA gene [20] was identified for the first time in an azithromycin-treated yaws patient; the mutation had spread to additional epidemiologically related persons. As expected, this mutation was present in both 23S rRNA genes of the TPE isolate [39]. In a previous study, the emergence of the A2059 mutation [21] was described in a yaws patient [40]. It is unknown how many people in this study were infected with yaws at the time of azithromycin administration, which was given to over 56 thousand people. Based on the number of TPE-positive samples (n = 302), we can estimate that at least several hundred yaws-infected were treated with azithromycin. Previous work predicted the probability of *de novo* emergence of macrolide resistance per treated patient to be 10^−2^ or lower [41,42], and this work provides a more accurate prediction in the range of 3×10^−3^ or lower.

The TP0136 and TP0326 loci were amplified and sequenced from 235 samples used in this study to compare the results with the TPE typing scheme published by Godornes et al. (2017) [11]. Both typing schemes showed similar typing resolution (Table 4) except for a single strain, where the previously developed typing scheme [11] identified TPE genotype S_E_22 with an unexpected TP0136 allele. The new TP0136 allele (described as U in this study) again emerged by novel intragenomic recombination events not yet described in TPE and reflected the modular structure of TPE TP0136 [18].

The limitations of this study include the fact that samples originated in a size- and geographically-limited population located in South Pacific Islands, having a low genetic diversity of infecting TPE bacteria, which resulted in a limited performance of the applied typing scheme. Another limitation comes from the fact that TPE genomes of African and Indonesian origin were used for selection of typing loci, potentially providing low resolution for isolates from South Pacific Islands.

The newly developed MLST typing scheme of TPE detected in human yaws isolates revealed a surprisingly low level of genetic diversity among TPE isolates infecting people in the Namatanai District of Papua New Guinea, consistent with the endemic nature of yaws. Two new alleles (one in TP0858 and one in TP0136) were shown to evolve by intragenomic recombination events by copying of paralogous sequences from other genomic regions via gene conversion (TP0858) or by deletion of modular sequences (TP0136).

## Supporting information

S1 Text **Table A**. Genome Sequences of TPE strains/isolates used for MLST target design. **Table B**. Genes/genomic regions excluded from the analyses (i.e., paralogous, and repetitive regions). **Table C**. Genes with the highest number of SNVs containing more than 8 SNVs per kbp among TPE strains/isolates. **Table D**. Characteristics of candidate genes considered for TPE typing. **Table E**. Primers used for the amplification of the first (outer primers) and the second step (inner primers) of nested PCR. **Table F**. Positively selected sites according to TPE Samoa D reference strain. **Fig A**. Phylogenetic tree based on sequences of the of typing loci including TP0488, TP0548, and TP0858.
(DOCX)
