# Supplementary material for: Low genetic diversity of Treponema pallidum ssp. pertenue (TPE) isolated from patients’ ulcers in Namatanai District of Papua New Guinea: Local human population is infected by three TPE genotypes
Source: PLoS Negl Trop Dis. 2024 Jan 2;18(1):e0011831. doi: 10.1371/journal.pntd.0011831 (PMC10786373; doi:10.1371/journal.pntd.0011831)
Supplement: S1 Text — Table A. Genome Sequences of TPE strains/isolates used for MLST target design. Table B. Genes/genomic regions excluded from the analyses (i.e., paralogous, and repetitive regions). Table C. Genes with the highest number of SNVs containing more than 8 SNVs per kbp among TPE strains/isolates. Table D. Characteristics of candidate genes considered for TPE typing. Table E. Primers used for the amplification of the first (outer primers) and the second step (inner primers) of nested PCR. Table F. Positively selected sites according to TPE Samoa D reference strain. Fig A. Phylogenetic tree based on sequences of the of typing loci including TP0488, TP0548, and TP0858. (DOCX) [file pntd.0011831.s001.docx]

**Supplementary Methods and Analysis**

**Low genetic diversity of Treponema pallidum ssp. pertenue (TPE) isolated from patients’ ulcers in Namatanai District of Papua New Guinea: local human population is infected by three TPE genotypes.**

Monica Medappa^1^, Petra Pospíšilová^1^, Maria Paula M.Madruga^1^, Lucy N. John^2,3^, Camila G. Beiras^3^, Linda Grillová^1^, Jan Oppelt^4^, Arka Banerjee^5^, Marti Vall-Mayans^6^, Oriol Mitjà^6,7,8^, David Šmajs^1*^

^1^Department of Biology, Faculty of Medicine, Masaryk University, Brno, Czech Republic

^2^National Department of Health, Aopi Centre, Port Moresby, Papua New Guinea

^3^Faculty of Medicine, University of Barcelona, Barcelona, Spain

^4^Department of Pathology and Laboratory Medicine, University of Pennsylvania, United States of America

^5^Department of Mathematics and Statistics, Indian Institute of Technology, Kanpur, Uttar Pradesh, India

^6^Skin NTDs and STI section, Fight Infectious Diseases Foundation, University Hospital Germans Trias i Pujol, Badalona, Spain

^7^Lihir Medical Centre, Lihir Island, Papua New Guinea

^8^School of Medicine and Health Sciences, University of Papua New Guinea, Port Moresby, Papua New Guinea

^*^Correspondence to: Prof. David Šmajs, Faculty of Medicine, Masaryk University, 625 00 Brno, Czech Republic, Phone: +420 549 497 496, [dsmajs@med.muni.cz](mailto:dsmajs@med.muni.cz)

**Analysis and selection of MLST typing loci**

Prior to this study, we analyzed 23 available complete and draft genome sequences of TPE isolated from human or NHP clinical materials from Africa and Pacific regions and identified candidate typing loci suitable for TPE molecular typing. Candidate loci for TPE typing were identified in the set of TPE genome sequences comprising 10 whole and 13 draft genome sequences available in 2018 at the time of the study design (Table A). These fulfilled our inclusion criteria requiring a minimum of 85% genome breadth of coverage covered by at least three good-quality reads. The genome sample set (n=23) represents TPE strains/isolates collected in Africa, Indonesia, and Oceania between 1953 and 2013 and were collected from humans and nonhuman primates. Given the limited stability of *tpr*E, G, and J [1], we excluded *tpr* genes from the analyses. In addition, other paralogous regions, genes with repetitions (Table B), and intergenic regions were also excluded. Analyzed genomes were aligned to the TPE Samoa D reference genome with *nucmer* using the MUMmer package [2]. The same tool was used to determine genome alignment-based SNPs. SNPs from all genome alignments were merged, and variable locations were determined in R (R Core Team, 2017) with packages *dplyr* [3], *rio* [4], and *Bioconductor* [5], *Biostrings* [6], and *rtracklayer* [7]. NCBI GenBank gene annotation was used to determine SNPs located in genes. Frequencies of SNPs were determined separately for genes present on + and − strands. The number of SNPs was divided by gene length and normalized to 1,000 bp (1 kbp) for each gene. Using this approach, we identified the most variable genes with the highest numbers of accumulated single nucleotide variants (SNVs) in a short DNA region (genes containing the highest SNVs frequency per kbp). Six candidate genes with SNVs frequency per kbp ranging from 8–29 were identified, which code for outer membrane proteins or hypothetical proteins (TP0136, TP0326, TP0488, TP0548, TP0858, TP0865; Table C). Most of these loci were previously identified as genes undergoing putative intra- or inter-strain recombination events in individual TP strains and also sequences evolving under positive selection [8][9][10].

**Table A.** Genome sequences of TPE strains/isolates used for MLST target design

| **Strains ID** | **Genome** | **Source** | **Host** | **Year of isolation** | **Geographic area** | **References** |
| --- | --- | --- | --- | --- | --- | --- |
| CDC-1 | Complete | Rabbit inoculation^1^ | human | 1980 | Ghana | Accession CP024750 |
| CDC-2 | Complete | Rabbit inoculation^1^ | human | 1980 | Ghana | [11] |
| Samoa D | Complete | Rabbit inoculation^1^ | human | 1953 | Western Samoa | [11] |
| Gauthier | Complete | Rabbit inoculation^1^ | human | 1960 | Congo | [11] |
| Fribourg-Blanc | Complete | Rabbit inoculation^1^ | NHPs | 1966 | Ghana | [12] |
| CDC2575 | Complete | Rabbit inoculation^1^ | human | 1980 | Ghana | [13] |
| Ghana-051 | Complete | Rabbit inoculation^1^ | human | 1988 | Ghana | [13] |
| Sei Geringing | Complete | Rabbit inoculation^1^ | human | 1990 | Indonesia | [13] |
| Kampung Dallan | Complete | Rabbit inoculation^1^ | human | 1990 | Indonesia | [13] |
| LMNP-1 | Complete | Clinical^2^ | NHPs | 2007 | Tanzania | [14] |
| LMN-2 | Draft | Clinical^2^ | NHPs | 2007 | Tanzania | [14] |
| Gambia-1 | Draft | Clinical^2^ | NHPs | 2014 | Gambia | [14] |
| Gambia-2 | Draft | Clinical^2^ | NHPs | 2014 | Gambia | [14] |
| Senegal NKNP-1 | Draft | Clinical^2^ | NHPs | 2015 | Senegal | [14] |
| Senegal NKNP-2 | Draft | Clinical^2^ | NHPs | 2015 | Senegal | [14] |
| Cote dIvoire TaiNP-1 | Draft | Clinical^2^ | human | 2013 | Ivory Coast | [14] |
| Cote dIvoire TaiNP-2 | Draft | Clinical^2^ | human | 2013 | Ivory Coast | [14] |
| ERR1470330 | Draft | Clinical^2^ | human | 2013 | Solomon Islands | [15] |
| ERR1470331 | Draft | Clinical^2^ | human | 2013 | Solomon Islands | [15] |
| ERR1470334 | Draft | Clinical^2^ | human | 2013 | Solomon Islands | [15] |
| ERR1470338 | Draft | Clinical^2^ | human | 2013 | Solomon Islands | [15] |
| ERR1470343 | Draft | Clinical^2^ | human | 2013 | Solomon Islands | [15] |
| ERR1470344 | Draft | Clinical^2^ | human | 2013 | Solomon Islands | [15] |

^1^ The TPE strain was multiplied in experimental animals prior to the genome sequencing.

^2^ Culture-independent enrichments (hybridization captures) of TP DNA were used before genome sequencing.

| **Gene** | **Product** | **Coordinates**^1^ |
| --- | --- | --- |
| TP_0117 | Tpr protein C | 134 912 – 136 708 |
| TP_0131 | Tpr protein D | 152 314 – 154 110 |
| TP_0313 | Tpr protein E | 329 144 – 331 432 |
| TP_0316 | Tpr protein F | 332 299 – 333 493 |
| TP_0317 | Tpr protein G | 333 552 – 335 822 |
| TP_0620 | Tpr protein I | 672 656 – 674 485 |
| TP_0621 | Tpr protein J | 674 543 – 676 819 |
| TP_0897 | Tpr protein K | 975 921 – 977 441 |
| TP_0225-226 | rRNA operon1 | 231 071 – 236 224 |
| TP_0265-267 | rRNA operon2 | 279 428 – 284 713 |
| TP_0136-138 | *tpr*K donor sites | 157 943 – 159 684 |
| TP_0433 | *arp*, acidic repeat protein | 461 695 - 463509 |
| TP_0470 | TPR domain protein | 498 736 – 499 845 |

**Table B.** Genes/genomic regions excluded from the analyses (i.e., paralogous, and repetitive regions)

^1^ Coordinates according to the Nichols genome (GenBank Acc. No. CP004010.2).

**Table C**. Genes with the highest number of SNVs containing more than 8 SNVs per kbp among TPE strains/isolates.

| **Locus^*^** | **Length (nt)** | **Protein function** | **No. of variable nt sites** | **SNVs frequency/kbp** |
| --- | --- | --- | --- | --- |
| TP0136 | 1412 | Fibronectin-binding protein | 41 | 29.04 |
| TP0548 | 1298 | Rare outer membrane protein | 36 | 27.73 |
| TP0858 | 1229 | Hypothetical protein | 33 | 26.85 |
| TP0488 | 2537 | Methyl-accepting chemotaxis protein | 31 | 12.22 |
| TP0865 | 1445 | Putative outer membrane protein | 26 | 17.99 |
| TP0326 | 2502 | Other membrane protein | 22 | 8.40 |

^*^Genes are listed according to the detected number of variable sites. Annotation and length of the genes were identified according to the yaws reference genome Samoa D (GenBank Acc. No. CP002374.1).

In the second step, we compared the resolution power of phylogenetic trees based on whole genome data and phylogenetic trees based on sequences of individual genes. Maximum likelihood (ML) phylogenetic trees were generated with MEGA 6 [16] using the Tamura Nei model [17] and 1000 pseudorandom bootstrap replicates. Deletions and insertions were counted as single events. Sequence Matrix 1.8 software was used for sequence concatenations [18]. R-studio and mEMBOSS [19] were used for further bioinformatics analyses.

A genome-wide ML phylogenetic tree from 23 available complete and draft genome sequences was based on 1,207 variable sites and could distinguish 22 haplotypes leading to identification of 6 main candidates (Table C). Since strains CDC2575 and Ghana-051 are completely identical [20], the genome-wide tree had 100% resolution of whole genome sequences. Among six candidate loci, the highest resolution power was observed for TP0488 (70%), followed by TP0326, TP0548, and TP0858 (each 57%), TP0136 (44%), and TP0865 (31.8%). Interestingly, the concatenated sequences of all candidate loci failed to reveal a higher resolution than the resolution observed in the TP0488 gene only (70%). Since previous studies on MLST of TPA revealed that amplification efficiency inversely correlates with the length of amplified regions [21], we aimed to select loci with the highest concentration of variable sites accumulated in a short DNA region (Table D).

Several selection criteria were therefore applied to obtain the most suitable loci for TPE typing, and these included (1) the percentage of genome-wide data resolution, (2) the number of SNVs per kbp, (3) the ability to distinguish TPE from TPA/TEN and TPE from TPA and TEN (Table D). With the approach described above, we propose a new MLST scheme for TPE strains based on sequencing three variable loci (TP0488, TP0548, and TP0858). This typing scheme can reveal 70% of the whole genome resolution using a 3,111 bp-long concatemer of variable regions and can distinguish TPA, TPE, and TEN infections. Additional analyses of the 23S rRNA genes for macrolide resistance/sensitivity evaluation are recommended.

| **Gene^*/**^** | **Variable region length** | **Variable region coordinates^**^** | **Percentage of genome-wide data resolution** | **Able to discern TPE from TPA/TEN^***^** | **Able to discern TPA/TPE/TEN^***^** |
| --- | --- | --- | --- | --- | --- |
| TP0488 | 782 bp | 522 942 – 523 723 | 70% | Yes | No |
| TP0326 | 2086 bp | 346 066 – 348 151 | 57% | Yes | No |
| TP0548 | 755 bp | 593 318 – 594 072 | 57% | No | No |
| TP0858 | 824 bp | 936 118 – 936 941 | 57% | Yes | Yes |
| TP0136 | 910 bp | 157 823 – 158 733 | 44% | Yes | Yes |
| TP0865 | 897 bp | 945 224 – 946 121 | 31.8% | Yes | Yes |

**Table D.** Characteristics of candidate genes considered for TPE typing.

^*^Genes are listed according to the percentage of genome-wide data resolution. ^**^According to the yaws reference genome Samoa D (GenBank Acc. No. CP002374.1). ^***^To identify TPA/TPE/TEN subspecies, TPE genomes listed in Table A were used. Representatives of TEN (Bosnia A, GenBank Acc. No. CP007548.1) and TPA (Nichols, GenBank Acc. No. CP004010.2; SS14, GenBank Acc. No. CP004011.1; Mexico A, GenBank Acc. No. CP003064.1) were used as well.

Based on the described analysis, primers used for amplification were designed and tested. Sequences of the primers used for the first (outer primers) and the second step PCR (inner primers) are shown in Table E.

**Table E.** Primers used for the amplification of the first (outer primers) and the second step (inner primers) of nested PCR.

| Locus | Outer primer | Inner primer | Length of PCR inner product |
| --- | --- | --- | --- |
| TP0488 |  |  | 1117 bp |
| Forward | 5' CGGAGCTTTTTCCCGTAA 3' | 5' TGGGTGAAGGGTCTTGTGAC 3' |  |
| Reverse | 5' AGCAAAGCGAATCTTCTCCA 3' | 5' GATACCTCGTCCCCTCCAAC 3' |  |
| TP0548 |  |  | 1018 bp |
| Forward | 5' TGGGGCACTAAACCGGAAGA 3' | 5´ GCGGTCCCTATGATATCGTGT 3´ |  |
| Reverse | 5' TACGGGCATTTGCGGATAGG 3' | 5´ GAGCCACTTCAGCCCTACTG 3´ |  |
| TP0858 |  |  | 976 bp |
| Forward | 5' CTGCTCGGACGCAAGTAAAG 3' | 5' CATTACAATGGGCACCATGA 3' |  |
| Reverse | 5' ACTCCCACTCGCATGTTAGC 3' | 5' AGCTCGAACTCAAGCTCAGG 3' |  |

**Fig A.** Phylogenetic tree based on sequences of the of typing loci including TP0488, TP0548, and TP0858. The TPE isolates from non-human primate origin (taken from the study by Janečková *et al*., [22]) clustered somewhat separately from human isolates. Note that J_E_11 genotype is very similar to TPE Kampung-Dalan K363 strain as they share the same recombinant allele in TP0858 locus [13]. The evolutionary history was inferred by using the Maximum Likelihood method based on the Tamura-Nei model. The percentage of trees in which the associated taxa clustered together is shown next to the branches. There was a total of 3042 positions in the final dataset.





**Table F**. Positively selected sites according to TPE Samoa D reference strain.

The MLST loci of the typing scheme used in this study were utilized to determine the presence of codons that displayed positive selection using the algorithm CODEML of the PAML package [10]. The specific loci analysed were TPESAMD_0488 (1116 bp, 372 codons), TPESAMD_0548 (1017 bp, 339 codons) and TPESAMD_0858 (972 bp, 324 codons) according to TPE Samoa D reference strain.

The positively selected sites for TPESAMD_0488, TPESAMD_0548 and TPESAMD_0858 are summarized in the table below.

| TPESAMD_0488 |
| --- |

| Amino acid position (TPESAMD_0488) | Amino acid  (TPESAMD_0488) | Probability (Pr)  (w>1) |
| --- | --- | --- |
| 186 | I | 1.000** |
| 203 | I | 0.981* |
| 233 | A | 0.982* |
| 239 | E | 0.981* |
| 274 | K | 0.982* |
| 296 | D | 0.998** |
| 297 | D | 0.981* |
| 300 | I | 0.998** |
| 318 | A | 0.998** |
| 333 | F | 0.981* |
| 405 | I | 0.981* |
| 417 | T | 0.982* |
| 445 | V | 0.981* |

| TPESAMD_0548 |
| --- |

| Amino acid position (TPESAMD_0548) | Amino acid (TPESAMD_0548) | Probablity (Pr)  (w>1) |
| --- | --- | --- |
| 48 | A | 0.953* |
| 49 | A | 0.953* |
| 50 | L | 1.000** |
| 53 | E | 1.000** |
| 54 | S | 0.953* |
| 55 | N | 1.000** |
| 56 | S | 1.000** |
| 57 | N | 0.996** |
| 58 | D | 0.996** |
| 59 | N | 0.996** |
| 145 | T | 0.996** |
| 159 | S | 0.996** |
| 160 | N | 0.996** |
| 161 | K | 1.000** |
| 162 | S | 0.996** |
| 163 | G | 0.953* |
| 164 | G | 1.000** |
| 165 | H | 0.953* |
| 204 | K | 0.952* |
| 206 | K | 0.996** |
| 208 | G | 0.996** |
| 252 | N | 1.000** |
| 253 | D | 0.996** |
| 254 | S | 0.996** |
| 255 | E | 0.954* |
| 314 | K | 0.996** |
| 317 | V | 0.996** |

| TPESAMD_0858 |
| --- |

| Amino acid position (TPESAMD_0858) | Amino acid (TPESAMD_0858) | Probablity (Pr)  (w>1) |
| --- | --- | --- |
| 146 | T | 0.965* |
| 148 | E | 0.957* |
| 149 | T | 0.969* |
| 278 | C | 0.994** |
| 279 | E | 1.000** |
| 280 | D | 0.999** |
| 281 | K | 0.966* |
| 284 | V | 0.997** |
| 285 | Y | 0.958* |
| 323 | K | 0.956* |
| 327 | E | 0.962* |

**References**

1. Mikalová L, Pospíšilová P, Woznicová V, Kuklová I, Zákoucká H, Smajs D. Comparison of CDC and sequence-based molecular typing of syphilis treponemes: tpr and arp loci are variable in multiple samples from the same patient. BMC Microbiol. 2013;13: 178. doi:10.1186/1471-2180-13-178
2. Kurtz S, Phillippy A, Delcher AL, Smoot M, Shumway M, Antonescu C, et al. Versatile and open software for comparing large genomes. Genome Biology. 2004;5: R12. doi:10.1186/gb-2004-5-2-r12
3. Wickham H, Francois R, Henry L, Müller K. dplyr: A grammar of data manipulation, 2015. URL https://github. com/hadley/dplyr. version 0.1.[p 1]. 2017
4. Chung-hong Chan, Geoffrey CH Chan, Thomas J. Leeper, and Jason Becker. rio: A Swiss-army knife for data file I/O. R package version 0.5.0. 2017.
5. Huber W, Carey VJ, Gentleman R, Anders S, Carlson M, Carvalho BS, et al. Orchestrating high-throughput genomic analysis with Bioconductor. Nat Methods. 2015;12: 115–121. doi:10.1038/nmeth.3252
6. Pagès H, Aboyoun P, Gentleman R, DebRoy S. Biostrings: efficient manipulation of biological strings. R package version 2.46.0.2017.
7. Lawrence M, Gentleman R, Carey V. rtracklayer: an R package for interfacing with genome browsers. Bioinformatics. 2009 Jul 15;25(14):1841–2.
8. Noda AA, Méndez M, Rodríguez I, Šmajs D. Genetic Recombination in Treponema pallidum: Implications for Diagnosis, Epidemiology, and Vaccine Development. Sex Transm Dis. 2022;49: e7–e10. doi:10.1097/OLQ.0000000000001497
9. Pla-Díaz M, Sánchez-Busó L, Giacani L, Šmajs D, Bosshard PP, Bagheri HC, et al. Evolutionary Processes in the Emergence and Recent Spread of the Syphilis Agent, Treponema pallidum. Mol Biol Evol. 2021;39: msab318.doi:10.1093/molbev/msab318
10. Maděránková D, Mikalová L, Strouhal M, Vadják Š, Kuklová I, Pospíšilová P, et al. Identification of positively selected genes in human pathogenic treponemes: Syphilis-, yaws-, and bejel-causing strains differ in sets of genes showing adaptive evolution. PLoS Negl Trop Dis. 2019;13: e0007463. doi: 10.1371/journal.pntd.0007463
11. Čejková D, Marie Zobaníková, Chen L, Pospíšilová P, Strouhal M, Qin X, et al. Whole Genome Sequences of Three Treponema pallidum ssp. pertenue Strains: Yaws and Syphilis Treponemes Differ in Less than 0.2% of the Genome Sequence. PLOS Neglected Tropical Diseases. 2012;6: e1471. doi:10.1371/journal.pntd.0001471
12. Zobaníková M, Strouhal M, Mikalová L, Čejková D, Ambrožová L, Pospíšilová P, et al. Whole Genome Sequence of the Treponema Fribourg-Blanc: Unspecified Simian Isolate Is Highly Similar to the Yaws Subspecies. PLOS Neglected Tropical Diseases. 2013;7: e2172. doi:10.1371/journal.pntd.0002172
13. Strouhal M, Mikalová L, Haviernik J, Knauf S, Bruisten S, Noordhoek GT, et al. Complete genome sequences of two strains of Treponema pallidum subsp. pertenue from Indonesia: Modular structure of several treponemal genes. PLOS Neglected Tropical Diseases. 2018;12: e0006867. doi: 10.1371/journal.pntd.0006867
14. Knauf S, Gogarten J, Schuenemann V, De Nys H, Düx A, Strouhal M, et al. African nonhuman primates are infected with the yaws bacterium Treponema pallidum subsp. pertenue. bioRxiv. 2017. doi:10.1101/135491
15. Marks M, Fookes M, Wagner J, Butcher R, Ghinai R, Sokana O, et al. Diagnostics for Yaws Eradication: Insights From Direct Next-Generation Sequencing of Cutaneous Strains of Treponema pallidum. Clin Infect Dis. 2018;66: 818–824. doi:10.1093/cid/cix892
16. Tamura K, Stecher G, Peterson D, Filipski A, Kumar S. MEGA6: Molecular Evolutionary Genetics Analysis Version 6.0. Mol Biol Evol. 2013;30: 2725–2729. doi:10.1093/molbev/mst197
17. Tamura K, Nei M. Estimation of the number of nucleotide substitutions in the control region of mitochondrial DNA in humans and chimpanzees. Mol Biol Evol. 1993;10: 512–526. doi: 10.1093/oxfordjournals.molbev.a040023
18. Vaidya G, Lohman D, Meier R. SequenceMatrix: Concatenation software for the fast assembly of multi-gene datasets with character set and codon information. Cladistics. 2010;27: 171–180. doi:10.1111/j.1096-0031.2010. 00329.x
19. Rice P, Longden I, Bleasby A. EMBOSS: The European Molecular Biology Open Software Suite. Trends in Genetics. 2000;16: 276–277. doi:10.1016/S0168-9525(00)02024-2
20. Strouhal M, Mikalová L, Havlíčková P, Tenti P, Čejková D, Rychlík I, et al. Complete genome sequences of two strains of Treponema pallidum subsp. pertenue from Ghana, Africa: Identical genome sequences in samples isolated more than 7 years apart. PLoS Negl Trop Dis. 2017;11: e0005894. doi: 10.1371/journal.pntd.0005894
21. Grillová L, Pĕtrošová H, Mikalová L, Strnadel R, Dastychová E, Kuklová I, et al. Molecular typing of Treponema pallidum in the Czech Republic during 2011 to 2013: increased prevalence of identified genotypes and of isolates with macrolide resistance. J Clin Microbiol. 2014;52: 3693–3700. doi:10.1128/JCM.01292-14
22. Janečková K, Roos C, Fedrová P, Tom N, Čejková D, Lueert S, Keyyu JD, Chuma IS, Knauf S, Šmajs D. The genomes of the yaws bacterium, *Treponema pallidum* subsp. *pertenue*, of nonhuman primate and human origin are not genomically distinct. PLOS Neglected Tropical Diseases. 2023;17: e0011602. doi: 10.1371/journal.pntd.0011602.
